# Editorial: Diabetes and oral health

**DOI:** 10.3389/fcdhc.2026.1786239

**Published:** 2026-01-23

**Authors:** Zoe Xiaofang Zhu, Thomas E. Van Dyke

**Affiliations:** 1 Department of Basic & Clinical Translational Science, Tufts University School of Dental Medicine, Boston, MA, United States; 2 ADA Forsyth Institute, Somerville, MA, United States; 3 Harvard School of Dental Medicine, Boston, MA, United States

**Keywords:** bidirectional relationship, clinical impact, diabetes mellitus, metabolic markers, periodontitis, therapeutic potentials

The bidirectional relationship between diabetes mellitus and oral health represents one of the most compelling examples of the oral health-systemic health connection. This Research Topic, “Diabetes and Oral Health,” assembles eight articles from 47 authors across multiple disciplines, exploring the mechanisms linking oral inflammation to systemic metabolism, therapeutic interventions, and clinical implications for integrated care (**Figure 1**). With over 32,000 views and 6,591 downloads, these contributions address a critical knowledge gap at the intersection of endocrinology and oral health.

**Figure 1:**
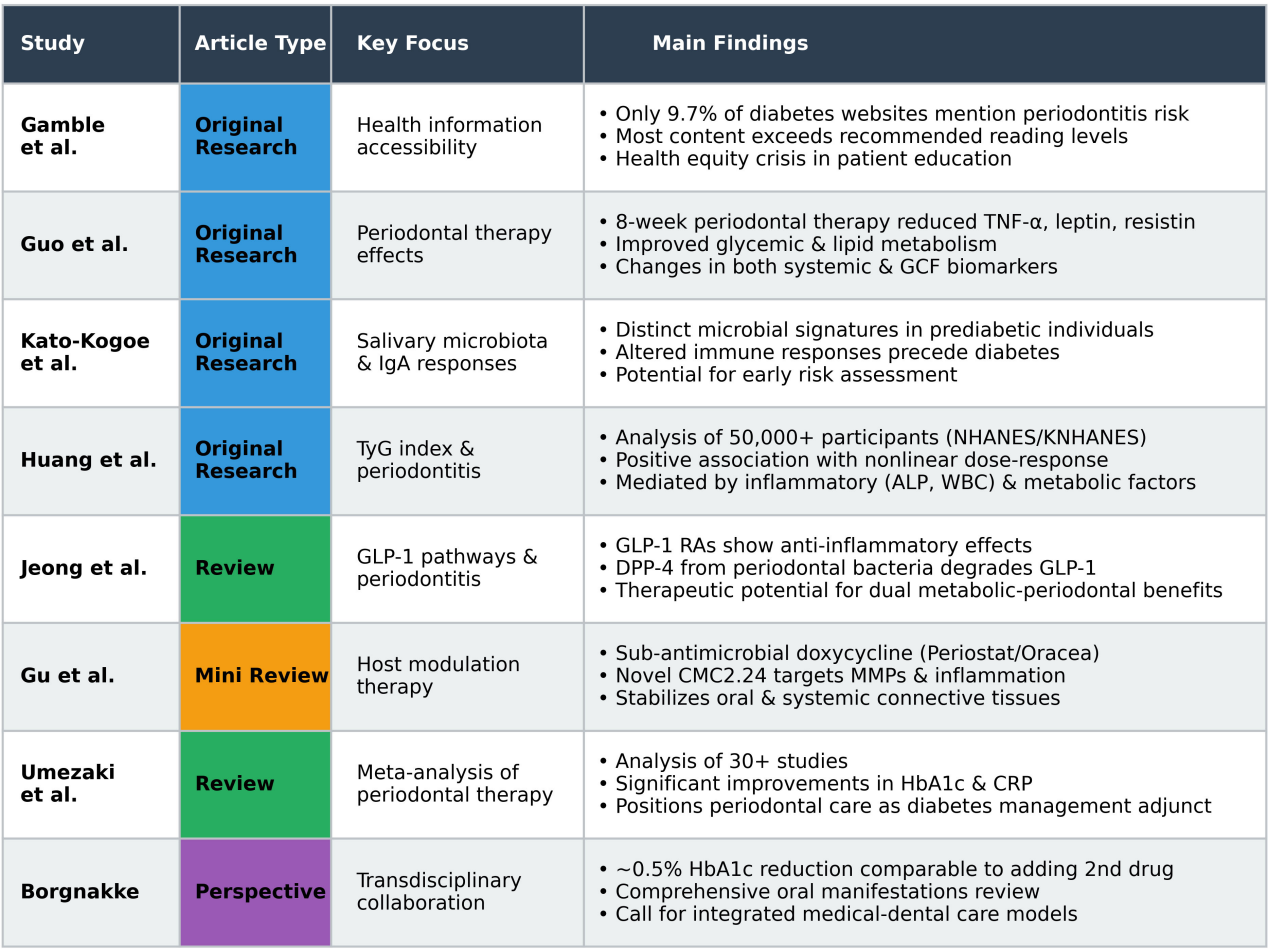
Summary of the articles comprising the “*Diabetes and Oral Health*” Research Topic. The collection includes four original research studies (blue), three reviews (green and orange), and one perspective article (purple), representing contributions from 47 authors. Original research articles examine health information accessibility (Gamble et al.), therapeutic effects of periodontal treatment (Guo et al.), salivary microbiota in prediabetes (Kato-Kogoe et al.), and metabolic risk markers (Huang et al.). Review articles synthesize evidence on GLP-1 pathways (Jeong et al.), host modulation therapy (Gu et al.), and meta-analytic findings on periodontal therapy outcomes (Umezaki et al.). The perspective article (Borgnakke) provides a comprehensive overview of oral manifestations in diabetes and advocates for transdisciplinary collaboration.

## Understanding the bidirectional relationship

The conceptualization of diabetes and periodontitis as interconnected conditions has been empirically observed in the clinic for many years, but recently the biological basis for the interconnection has evolved substantially. Periodontal diseases, once viewed as localized infections, are now recognized as inflammatory diseases and potential drivers of systemic inflammation capable of influencing metabolic health. Conversely, the hyperglycemic environment characteristic of diabetes creates conditions that favor periodontal tissue destruction and impair healing responses.

Gamble et al. identified a striking gap in health information accessibility. Their readability assessment of diabetes-related websites revealed that only 9.7% acknowledge periodontitis as a diabetes risk factor, and most available information exceeds recommended reading levels. This health equity crisis particularly affects socioeconomically disadvantaged populations who bear disproportionate burdens of both conditions, highlighting an urgent need for accessible, evidence-based patient education materials.

## Therapeutic interventions and metabolic impact

The therapeutic value of periodontal treatment extends beyond oral health outcomes. Guo et al. demonstrated that non-surgical periodontal therapy in diabetic patients produces progressive improvements in both local and systemic inflammatory markers. Their study revealed significant reductions in TNF-α, leptin, resistin, and free fatty acids, coupled with increased adiponectin levels and improved glycemic control over an eight-week period. Importantly, these changes occurred both systemically and in gingival crevicular fluid, establishing the periodontal pocket as an active site of metabolic crosstalk.

These findings underscore a critical mechanistic insight: periodontal inflammation contributes to systemic metabolic dysregulation through inflammatory mediators that directly influence insulin sensitivity and lipid metabolism. The reduction of local inflammatory burden through periodontal therapy may therefore represent a modifiable intervention point for improving overall metabolic health in diabetic patients.

## Microbial and immunological dimensions

Kato-Kogoe et al. expanded the microbiological understanding beyond bacteria by characterizing salivary microbiota and IgA responses in prediabetic individuals. Their identification of distinct microbial signatures and altered immune responses in the prediabetic state suggests that oral microbial dysbiosis may precede overt diabetes development. This observation raises intriguing questions about whether oral microbial changes could serve as early warning signs or even contribute to diabetes pathogenesis.

The oral microbiome’s role in this bidirectional relationship extends beyond periodontal pathogens alone, encompassing broader microbial community dynamics that influence both local and systemic immune regulation. Understanding these complex interactions may reveal novel intervention targets and biomarkers for risk stratification.

## Metabolic markers and risk assessment

Huang et al. investigated the association between the triglyceride-glucose (TyG) index—a validated surrogate marker of insulin resistance—and periodontitis using data from two large population-based surveys (NHANES and KNHANES). Their analysis of over 50,000 participants revealed a significant positive association between elevated TyG index and periodontitis risk, with a nonlinear dose-response relationship. Importantly, mediation analyses identified that inflammatory factors (alkaline phosphatase, white blood cells) and metabolic factors (vitamin D, HDL cholesterol) partially mediate this association, suggesting that insulin resistance influences periodontal health through both inflammatory and metabolic pathways. This work exemplifies how readily accessible metabolic biomarkers could facilitate early identification of individuals at elevated risk for periodontitis, particularly those with metabolic disorders, and underscores the potential value of integrated screening approaches in dental and medical practice.

## Synthesis of clinical evidence and future frontiers

To complement the original research, three reviews in this Research Topic synthesize the current evidence base to guide future clinical practice. Jeong et al. explore a novel frontier by examining the interplay between periodontitis and glucagon-like peptide-1 (GLP-1) pathways. Their scoping review suggests that GLP-1 receptor agonists—widely used in modern diabetes management—may offer pleiotropic benefits for the periodontium by reducing inflammation and supporting tissue regeneration.

Recognizing the systemic “hyper-inflammatory” state of the diabetic host, Gu et al. discuss the efficacy of host modulation therapy (HMT). Their review highlights sub-antimicrobial dose doxycycline and introduces emerging third-generation compounds, such as chemically-modified curcumin (CMC2.24), which target pathologically elevated matrix metalloproteinases to stabilize both oral and systemic connective tissues.

The clinical necessity of integrating these findings into standard care is further solidified by Umezaki et al. and Borgnakke. In a meta-analysis of over 30 studies, Umezaki et al. confirm that non-surgical periodontal therapy significantly improves HbA1c and C-reactive protein (CRP) levels, positioning periodontal care as a vital non-pharmacological adjunct in diabetes management. Furthermore, the Perspective article by Borgnakke delivers a powerful call to action, noting that the ~0.5 percentage point reduction in HbA1c achieved through periodontal treatment is comparable to the effect of adding a second oral anti-diabetic drug to metformin. By emphasizing shared inflammatory pathways and the potential for reduced healthcare costs, Borgnakke provides a roadmap for a patient-centered model where medical and dental professionals no longer operate in silos but collaborate to reduce systemic complications.

## Conclusion

This Research Topic advances our understanding of the diabetes-oral health axis through diverse methodological and disciplinary lenses. Collectively, these articles demonstrate that the relationship is not merely associative but involves active biological crosstalk with profound clinical consequences. As healthcare systems increasingly move toward holistic, patient-centered models, the integration of oral health into diabetes management and the reciprocal inclusion of metabolic screening in dental settings represent both a scientific opportunity and a clinical imperative.

We thank all the authors, reviewers, and editorial staff who contributed to this Research Topic. We hope these works will catalyze further research, inform clinical guidelines, and ultimately improve the quality of life for the millions worldwide affected by these interconnected chronic diseases.

